# Laser Ultrasound Investigations of AlScN(0001) and AlScN(11-20) Thin Films Prepared by Magnetron Sputter Epitaxy on Sapphire Substrates

**DOI:** 10.3390/mi13101698

**Published:** 2022-10-09

**Authors:** Elena A. Mayer, Olga Rogall, Anli Ding, Akash Nair, Agnė Žukauskaitė, Pavel D. Pupyrev, Alexey M. Lomonosov, Andreas P. Mayer

**Affiliations:** 1 B + W Department, Offenburg University of Applied Sciences, 77652 Offenburg, Germany; 2 Fraunhofer Institute for Applied Solid State Physics IAF, 79108 Freiburg, Germany; 3 Fraunhofer Institute for Organic Electronics, Electron Beam and Plasma Technology FEP, 01277 Dresden, Germany; 4 School of Science, Nanjing University of Science and Technology, Nanjing 210094, China

**Keywords:** laser ultrasound, AlScN, surface acoustic waves, magnetron sputter epitaxy, elastic properties, thin films

## Abstract

The laser ultrasound (LU) technique has been used to determine dispersion curves for surface acoustic waves (SAW) propagating in AlScN/Al_2_O_3_ systems. Polar and non-polar Al_0.77_Sc_0.23_N thin films were prepared by magnetron sputter epitaxy on Al_2_O_3_ substrates and coated with a metal layer. SAW dispersion curves have been measured for various propagation directions on the surface. This is easily achieved in LU measurements since no additional surface structures need to be fabricated, which would be required if elastic properties are determined with the help of SAW resonators. Variation of the propagation direction allows for efficient use of the system’s anisotropy when extracting information on elastic properties. This helps to overcome the complexity caused by a large number of elastic constants in the film material. An analysis of the sensitivity of the SAW phase velocities (with respect to the elastic moduli and their dependence on SAW propagation direction) reveals that the non-polar AlScN films are particularly well suited for the extraction of elastic film properties. Good agreement is found between experiment and theoretical predictions, validating LU as a non-destructive and fast technique for the determination of elastic constants of piezoelectric thin films.

## 1. Introduction

Among the various fields of application of Al_1-x_Sc_x_N films, analog signal processing devices for mobile communication play a preeminent role. The working principle of such devices, such as frequency filters and delay lines, is based on bulk or surface acoustic waves (BAW and SAW, respectively). The main reason for the growing interest in AlScN films constitutes their favorable piezoelectric and mechanical properties [1,2,3]. Piezoelectric properties of this ternary nitride can be enhanced by increasing the scandium concentration x, which has consequences for the electromechanical coupling and also for the elastic properties and hence the velocities of acoustic waves [4,5,6]. For the design of signal processing devices containing AlScN films, knowledge of the elastic constants is of paramount importance. 

For the determination of elastic constants of AlScN films, a fast, non-destructive, and easy-to-handle method is called for. Optimally, such a method could also be employed for quality control of AlScN films. A particular challenge in the case of epitaxial AlScN films is the large number of material constants that have to be determined. The main goal of this study is to demonstrate that laser ultrasound (LU) is an attractive technique for this purpose. 

In the past, elastic constants of AlScN films have been determined with Brillouin scattering [7,8,9] and via electrical excitation of acoustic vibrations, especially with the help of SAW resonators (called resonator method in the following) [10,11] or delay lines [12] (for a review see for example [10]). However, preparing SAW or other types of resonators requires advanced fabrication capabilities to obtain the full dispersion curves from multiple test structures (phase velocity as a function of frequency).

The piezoelectric constants are yet out of reach for our laser ultrasound set-up. This problem is shared with Brillouin scattering, another non-destructive optical method, which (as with LU) does not require any specific modifications of the sample, unlike the resonator method. However, the LU approach needs much shorter measurement times, especially when being partially automated. A full SAW dispersion curve is obtained in one measurement cycle, taking about 30 min. The propagation direction on the surface can be varied without extra effort. This allows to efficiently exploit the anisotropy of the substrate to determine film properties from SAW dispersion curves, as was done here to investigate the highly anisotropic AlScN-on-sapphire systems. We show here that valuable information can be gained with comparatively robust and inexpensive instrumentation. For additional benchmarking of our experimental approach, a comparison to the results of ab initio calculations, using the density functional theory can be performed [5,13,14,15]. Here, we compare SAW dispersion curves obtained by LU to those calculated with the most recently published theoretical data on material constants of AlScN [5].

In the past, the determinations of elastic properties of thin films by laser ultrasound were mostly confined to isotropic films. (The only exception known to us are diamond films [16] and a pre-study of AlScN films [17]). 

In our previous study, we showed that LU can be used to characterize textured AlScN(0001) films on silicon Si(001) substrates [17]. In these samples, the acoustic pulses were generated by laser pulses at the interface between the transparent AlScN film and the opaque silicon substrate. The fact that sapphire substrates are also transparent to the laser light constitutes an additional challenge and requires a metal layer to absorb the laser pulse. More recently, in-plane oriented AlScN(0001) films were grown on sapphire Al_2_O_3_(0001) substrates by magnetron sputter epitaxy [18] and then used for SAW resonator fabrication. The motivation was two-fold: sapphire substrate enables higher phase velocity and higher frequency of SAW and at the same time the higher material quality leads to improved electromechanical coupling and quality factor Q [19]. In order to further increase electromechanical coupling k_eff_^2^, epitaxial a-plane (non-polar) AlScN(11-20) SAW structures were successfully achieved on r-plane Al_2_O_3_(1-102) [20,21]. By aligning the SAW propagation direction with the piezoelectric constant d_33_, an additional 85% improvement in k_eff_^2^ was demonstrated. In these earlier studies, a substrate off-cut angle was shown to strongly influence the crystalline quality of AlScN. As the ability to deposit high-quality non-polar AlScN is a rather recent discovery [22], no measurements of elastic constants have been reported so far. In this work we propose using LU to estimate the elastic properties of Al_0.77_Sc_0.23_N(11-20)/Al_2_O_3_(1-102), especially focusing on high anisotropy in this material system. 

The paper is organized in the following way. In Section 2, the geometries of the systems investigated in this work are defined and their fabrication process and characterization are described. Moreover, details of the LU technique, as applied to the investigation of AlScN films, are given. 

In the first part of Section 3, the results of our investigations of the elastic properties of the sapphire substrates are presented. This knowledge is a prerequisite for the determination of film properties by SAW-based LU. Values for the elastic moduli of sapphire were compiled from the literature. They were used as input for simulations of the SAW slowness curves of c-plane and r-plane sapphire. Simulated SAW velocities are compared with corresponding LU measurement results. 

Next, experimental data are presented for dispersion curves of SAW propagating in Al_0.77_Sc_0.23_N films. Both AlScN(0001)/Al_2_O_3_(0001) and AlScN(11-20)/Al_2_O_3_(1-102) were investigated. These first LU measurement results for AlScN films on sapphire are compared with simulated dispersion curves using the ab initio elastic and piezoelectric constants of [5]. Very good agreement is found between theory and experiment. 

In order to assess the significance of this agreement for the individual material constants of Al_0.77_Sc_0.23_N, a sensitivity analysis is presented for the SAW dispersion curves in the frequency range accessible for our LU setup for both geometries studied. 

## 2. Materials and Methods

### 2.1. Substrate and Film Geometries

**Figure 1:**
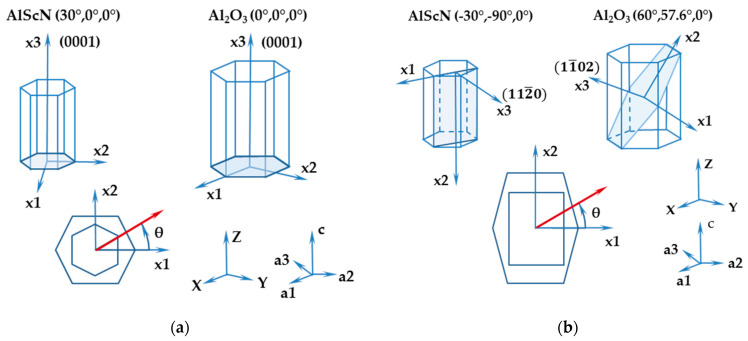
Crystal cuts of Al_2_O_3_ substrate and AlScN film, defined in the hexagonal crystal system (a1,a2,a3,c) and with the help of the two first Euler angles *λ* and *μ* (rotation with respect to Cartesian coordinate system XYZ). The third Euler angle *θ* defines the direction of SAW propagation (red arrows) on the surface planes (x1x2). (**a**) The c-plane (0001) cuts of both film and substrate; (**b**) a-plane AlScN(11-20) film on the r-plane Al_2_O_3_(1-102) substrate.

As already mentioned, for the LU investigations of elastic properties of AlScN films reported here, two different layered structures were considered, i.e., AlScN(0001)/Al_2_O_3_(0001) and AlScN(11-20)/Al_2_O_3_(1-102). In Figure 1, the epitaxial relationship between the film and substrate material is shown schematically for the two systems. The Cartesian coordinate systems introduced after rotation with the x_3_-axis normal to the surface correspond to the Euler angles (*λ*, *μ*, *θ*) = (0°, 0°, *θ*) for c-plane sapphire and (*λ*, *μ*, *θ*) = (60°, 57.6°, *θ*) for r-plane sapphire, respectively. The Euler angle *μ* = 57.6° for perfect r-plane orientation results from a ratio of lattice constants c/a = 2.73 for sapphire. For optimal growth conditions of the non-polar AlScN film, substrate off-cut angle *χ* is needed, which is related to the second Euler angle via *χ =* *μ* − 57.6°.

We note that the angle *θ* defines the wavevector direction of SAW propagating on the corresponding surface. 

### 2.2. Fabrication of AlScN Films

AlScN(0001)/Al_2_O_3_(0001) and AlScN(11-20)/Al_2_O_3_(1-102) thin films were grown by the magnetron sputter epitaxy method [18,20,22]. In the case of non-polar AlScN, 3° substrate off-cut was found to be the best for high crystalline quality [20], a detailed growth optimization study including a proposed growth model for non-polar III-nitrides and different off-cut angles is published elsewhere [22]. All films were grown on ⌀ = 100 mm substrates in an Evatec sputter cluster tool (base pressure ~5 × 10^−6^ Pa), using reactive pulsed-DC magnetron co-sputtering. Substrate rotation ensured the composition and thickness uniformity of the films. The scandium concentration x = 0.23 was achieved by setting the P(Al, 99.9995% pure) = 684 W and P(Sc, 99.99% pure) = 316 W. This specific Sc concentration was chosen as it allowed us to deposit AlScN thin films with very high crystalline quality and low density of abnormally oriented grains, leading to reliable evaluation of material properties by LU. Prior to deposition, the sapphire substrates were cleaned in-situ using Ar inductively coupled plasma (ICP) etching and the targets were pre-sputtered in Ar behind a closed shutter. More details about the growth conditions can be found in [18,20,22], all parameters except for the N_2_ gas flow were kept the same, as summarized in Table 1.

The scandium concentration (+/−2% error) was estimated using energy dispersive x-ray (scanning electron microscope Zeiss Auriga Crossbeam FIB-SEM with EDX spectroscopy from Bruker Quantax) on AlScN(0001)/Si(001) films deposited under the same conditions. This was done in order to avoid the overlap of Al emission peaks from the sapphire substrate and AlScN film [18]. No variation of composition was observed between the edge and the center of the wafers. The average film thickness (+/−3% error) was determined by spectroscopic ellipsometry (J.A. Woollam M-2000X), using a model optimized for AlScN from [23]. Thickness uniformity analysis indicated <3% variation in thickness across the wafer. The surface roughness of the films and the thickness of the metal layers were evaluated by atomic force microscopy (AFM, Bruker Dimension Icon) in tapping mode (not shown). The crystalline quality and in-plane orientation of AlScN films were confirmed by X-ray diffraction (XRD) [18,20] (not shown). Based on XRD pole figures analysis, the epitaxial relationship for c-plane AlScN could be defined as [10-10]_AlScN_//[11-20]_sapphire_ and (0001)_AlScN_//(0001)_sapphire_ and for a-plane AlScN [0001]_AlScN_//[1-101]_sapphire_ and [1-100]_AlScN_//[11-20]_sapphire_, respectively.

Details on the samples investigated by LU in this work are given in Table 2.

### 2.3. Application of the Laser Ultrasound Approach

**Figure 2:**
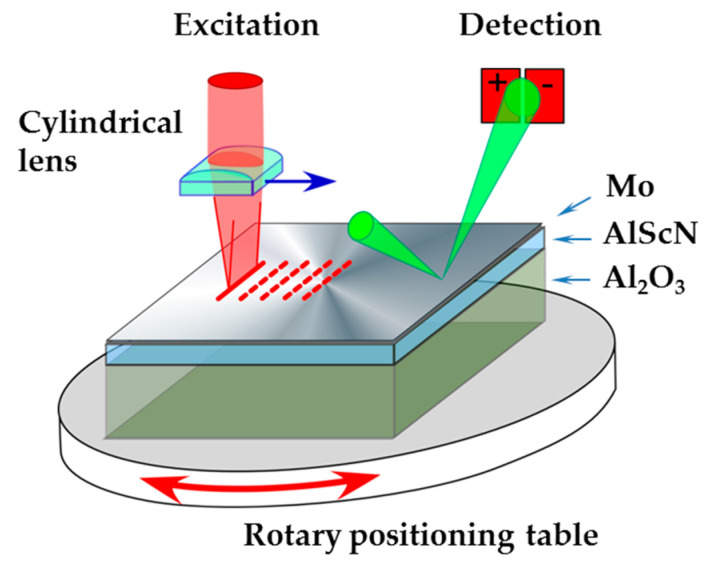
Experimental setup.

A schematic drawing of the LU set-up is shown in Figure 2. Acoustic pulses, traveling along the sample surface, are excited by laser pulses via the thermoelastic effect. This excitation mechanism is explained in detail in [24,25]. The optical pulses are generated by a passively Q-switched Nd:YAG laser (pulse duration: 1 ns; wavelength: 1064 nm, frequency-doubled to 532 nm). They are focused on a straight line on the surface with a cylindrical lens to minimize diffraction of the excited SAW and to achieve a well-defined wavevector direction. 

With the help of a continuous-wave laser, the acoustic surface pulses are detected with the probe-beam deflection method at a fixed observation point at the surface for various positions of the movable line source. Their shapes are recorded along with the distances of the observation points from the source. The presence of a film on the substrate surface causes the pulse shape to change with distance from the source. The quantity measured by probe-beam deflection at each observation point is the local surface slope as a function of time [25]. If the film and substrate are both transparent at the frequency of laser light used for the excitation of ultrasound pulses, the surface has to be coated with a thin metal film to ensure absorption of the laser pulses and enable thermoelastic excitation of SAW pulses. The effect of this additional layer has to be accounted for in the data interpretation and analysis. 

By Fourier decomposition of the pulse shapes at the different distances between the line source and observation point, the phase velocities of each individual Fourier component are determined. In this way, a full dispersion curve of SAW with wavevectors vertical to the line source is obtained in one measurement cycle. Using a translation stage with a controlled stepper motor, the measurements are partly automated. The broad-band character of the LU method and its semi-automated operation renders it a fast tool for the characterization of near-surface elastic properties of materials. The achievable frequency range is partly material-dependent with an upper limit of 400 to 600 MHz for the materials investigated here with our set-up. With the help of a rotary positioning table for the sample, SAW dispersion curves can be measured for any wavevector direction on the surface. 

Since even the third harmonic of the carrier frequency of the Nd:YAG pulse laser is below the absorption edges of sapphire and AlN, a thin absorbing layer is needed to enable thermoelastic excitation of acoustic waves. Test measurements have been carried out to find a suitable surface metallization Main criteria were signal quality, availability, and especially the influence of the metal coating on the SAW dispersion, which should be kept as small as possible. After careful consideration, molybdenum and titanium were identified as suitable metal coatings, but only data with Mo are shown here (Table 2). 

The thickness of the AlScN film, together with the frequency range of the average SAW phase velocity, and to some extent, the mode character, determine the minimal penetration depth of the SAW in the substrate. For a rough estimate, we assume that the displacement field associated with a SAW has an appreciable magnitude up to distances of a wavelength from the surface. Our investigations refer to samples with AlScN films with a thickness of ≤1000 nm and reached frequencies up to 600 MHz. This suggests that even at the upper edge of the frequency range accessible for our LU experiments, the SAWs penetrate deeply into the substrate and consequently, their dispersion is strongly influenced by the density and elastic constants of the substrate. Therefore, these quantities have to be known to a high precision in order to be able to extract the elastic properties of the film from the SAW dispersion curves. The determination of elastic constants of the sapphire using LU is described in Section 3.1.1 and the off-cut angle assessment in Section 3.1.2, respectively. 

For data processing and evaluation and the interpretation of the measurement results, simulations of dispersion curves and displacement fields have been carried out with a computer program based on a semi-analytic Greens function approach (see e.g., [26]). In these simulations, a time (t) and position (x) dependent traction vector

(1) $$T=\hat{k}\;T_{0}\exp\left[i\left(\omega t-k\cdot x\right)\right]$$

is applied to the surface, where k is a wavevector in the surface plane, $\hat{k}$ the unit vector pointing into the wavevector direction, ω/(2π) a frequency, and $T_{0}$ an arbitrary traction amplitude, respectively. 

## 3. Results

### 3.1. Substrates

#### 3.1.1. Elastic Constants of Sapphire Substrates

As mentioned in Section 2, for extraction of the elastic properties of AlScN films with thicknesses smaller than 1 µm from SAW dispersion curves in a frequency range below 600 MHz, the elastic moduli and the density of the substrate have to be known with high precision. In Table 3, data from the literature are compiled for Al_2_O_3_. In the case of *c*_12_, the values of the different sources are at variance by more than 3%, and the value of *c*_14_ by more than 7%, in addition to the problem of finding the correct sign for this constant, which was resolved in [27,28]. 

**Figure 3:**
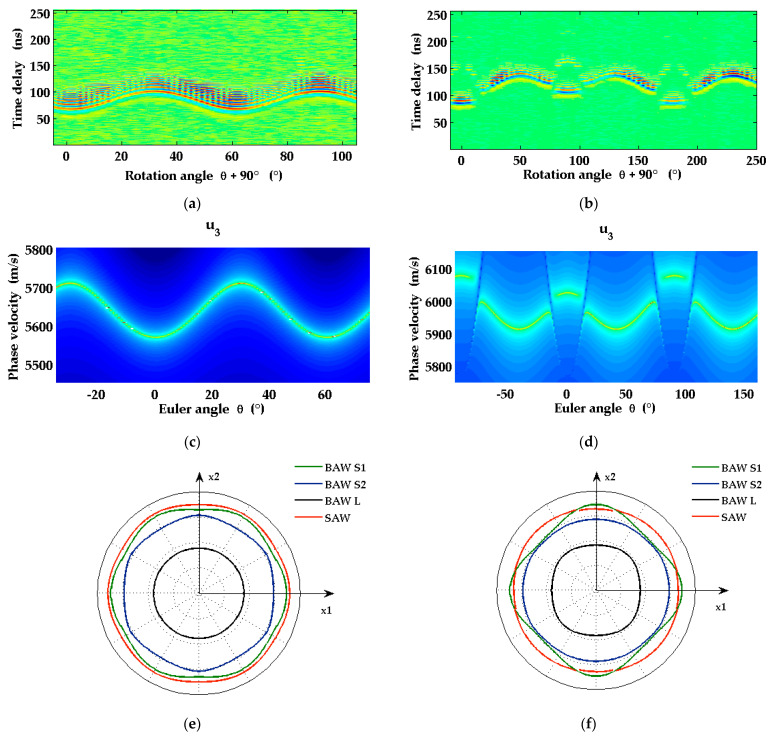
Dependencies on SAW wavevector direction for the sapphire cuts: c-plane Al_2_O_3_(0001) (**a**,**c**,**e**) with Euler angles (0°, 0°, *θ*) and r-plane Al_2_O_3_(1-102) (**b**,**d**,**f**) with Euler angles (60°, 57.6°, *θ*). Measured signal on the surface of sample 1 in Table 2 (**a**) and of sample 2 in Table 2 (**b**). Measurements taken at various angles using a rotary positioning table; (**c**,**d**) show the calculated SAW phase velocities on the surface of pure sapphire crystal as functions of Euler angle *θ*; (**e**,**f**) present the intersections of the slowness surface of bulk acoustic waves with the surface plane (quasi-longitudinal sheet (BAW L), two quasi-shear sheets (BAW S1 and BAW S2)) and the slowness curve for Rayleigh waves (SAW) on pure sapphire as functions of the Euler angle *θ*.

**Figure 4:**
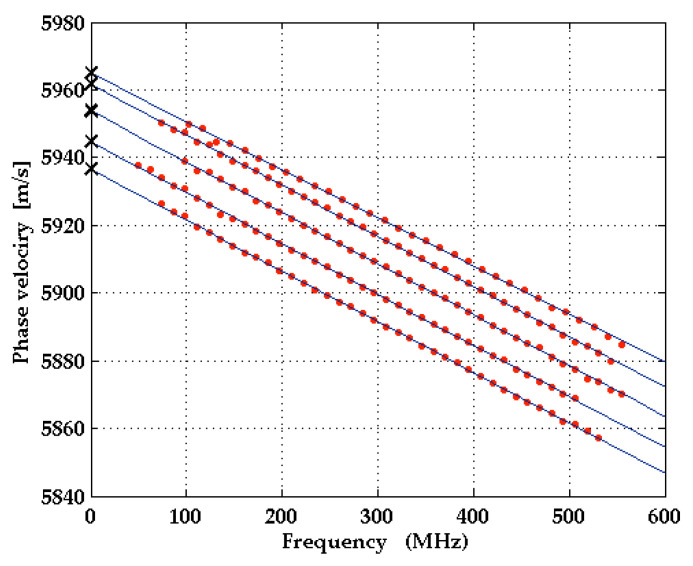
Experimental dispersion curves for the directions *θ* = 15°, 18°, 21°, 24°, 27° on r-plane sapphire with a 50 nm molybdenum film on top (Sample 2 in Table 2). Extrapolation of fitted straight lines to zero frequency gives the values of phase velocity (black crosses) on pure sapphire.

LU measurements were carried out on two samples with sapphire substrates in two different orientations, Al_2_O_3_(0001) (Sample 1 in Table 2) and Al_2_O_3_(1-102) (Sample 2 in Table 2). The surfaces of both samples were coated with a Mo layer of ~50 nm thickness. Sample 1 had an additional AlScN film of 1 µm thickness between the substrate and the Mo layer. Figure 3b shows the dependence of surface acoustic pulses in the Al_2_O_3_(1-102) sample, coated with a Mo layer only (Sample 2 in Table 2), detected at a fixed distance from the line source, on the wavevector direction (surface slope at observation point as a function of the arrival time and angle *θ*). In Figure 4, SAW dispersion curves obtained from the LU measurements on Sample 2 in Table 2 are shown for five different wavevector directions on the surface. Because of the small thickness of the metal layer in comparison to the SAW wavelength, the dispersion curves are essentially straight lines. The SAW phase velocity for the uncoated sapphire surface is obtained by extrapolating these straight lines to zero frequency. In this way, the dependence of the SAW phase velocity on the wavevector direction and hence the slowness curve of SAWs on this surface of sapphire can be determined experimentally. 

Figure 3d shows the results of simulations of the local surface displacement $u_{3}$ for Al_2_O_3_(1-102) as a response to a surface traction (1). Note that the quantity measured by probe-beam deflection in our LU setup is the local surface slope, i.e., the directional derivative of $u_{3}$ along the SAW wavevector. In a homogeneous medium with planar surface, this response depends on the frequency ω/(2π) and wavelength 2π/|k| of the excitation (surface traction in (1)) via the ratio ω/|k| only (phase velocity in Figure 3c,d). The SAW phase velocities correspond to the maxima of $\left|u_{3}\right|$ (bright curves emerging in Figure 3c,d). Comparison with Figure 3b confirms the expected inverse behavior of the SAW phase velocity and the delay time of the SAW pulses. 

The weakness of the detected signal in certain intervals of angle *θ*, for example in the neighborhood of *θ* = 12° and *θ* = 78° in Figure 3d, can be explained by the smallness of the out-of-plane displacement amplitude $\left|u_{3}\right|$ in these angular intervals. The unusual features in the dependence of the SAW phase velocity on the wavevector direction in the case of the r-plane surface are related to a phenomenon visible in Figure 3f. Here, the slowness curve of SAW propagating on the r-plane surface of sapphire is shown together with the intersection curves of the surface and the three sheets of the slowness surface of bulk acoustic waves (quasi-longitudinal, fast quasi-shear, and slow quasi-shear). In the angular regions corresponding to wavevector directions with faint signals (two at the same wavevector direction) and comparatively strong variation of the SAW phase velocity, the SAW slowness curve and the intersection curve of the sheet of slow quasi-shear bulk waves approach, and a repulsion of these two modes can be seen. Here, a transition occurs from the usual situation of the SAW velocity being the lowest phase velocity of acoustic modes for a given wavevector direction parallel to the surface, to an interval of wavevector directions where the phase velocity of SAW is larger than that of the slow quasi-shear bulk waves. This effect does not occur in the case of Al_2_O_3_(0001) (Figure 3c,e).

**Figure 5:**
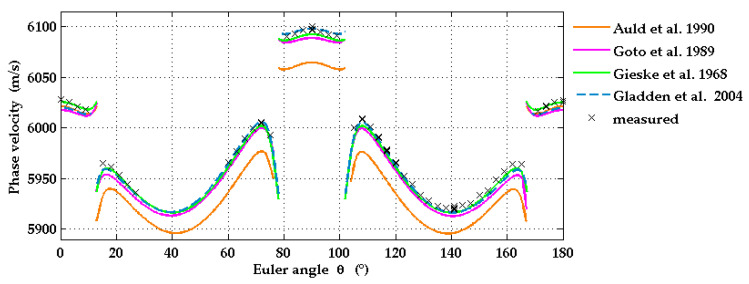
Theoretical dependencies of SAW phase velocities on wavevector direction for r-plane sapphire (solid and dashed lines) calculated using different sets of elastic constants taken from the literature (see Table 3), and compared with experimental data (black crosses).

The experimental results for the SAW phase velocities in Al_2_O_3_(1-102) were then compared with the results of calculations performed with the semi-analytic Greens function method. Input data for these calculations are the sets of elastic moduli listed in Table 3. For the density of sapphire, the value *ρ* = 3982 kg/m^3^ [31] was used, and for the dielectric constants we used the values $\varepsilon_{11}$ = 9.34 and $\varepsilon_{33}$ = 11.54 [33] in all our calculations. The off-cut angle of 3° was accounted for. It was found that the set of elastic constants reported by Gladden et al. [27] and also the constants provided by Gieske and Barsch [30], the latter after correction of the sign of *c*_14_, fit best to the LU data and lead to very good agreement between calculated and measured phase velocities. A comparison of calculated phase velocities with three different input sets and measured values as functions of wavevector direction is provided in Figure 5. 

**Figure 6:**
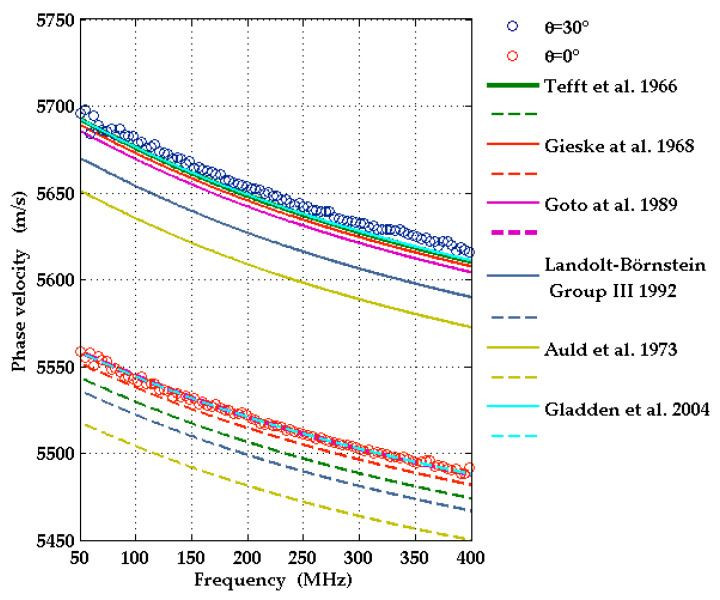
Experimentally obtained dispersion curves for the directions *θ* = 0° (red circles) and 30° (black circles) on the structure consisting of c-plane sapphire with a 1 µm thick Al_0.77_Sc_0.23_N film and a 50 nm molybdenum film on top, compared with corresponding simulated dispersion curves (solid and dashed lines for *θ* = 30° and for *θ* = 0°, respectively). The latter were obtained with material constants of sapphire from different authors (see Table 3).

This finding is confirmed by measurements and calculations of SAW dispersion curves for 1000 nm thick Al_0.77_Sc_0.23_N(0001)/Al_2_O_3_(0001) coated with ~50 nm Mo (Sample 1 in Table 2). Two different wavevector directions were considered. The dispersion due to the presence of the AlScN film leads to a broadening of the detected SAW pulse shapes (Figure 3a), and the dispersion curves in Figure 6 are no longer straight lines. In the calculations, the elastic, piezoelectric, and dielectric constants of [5] were used. The material constants of the molybdenum layer, *c*_11_ = 440 GPa, *c*_44_ = 126 GPa, and density *ρ* = 10,280 kg/m^3^, were taken from [34]. A comparison in Figure 6 shows very good agreement between experimental dispersion curves and those calculated with the elastic constants of sapphire taken from [30] with the corrected sign of *c*_14_.

#### 3.1.2. Off-Cut Angle in Al_2_O_3_(1-102) Substrates

**Figure 7:**
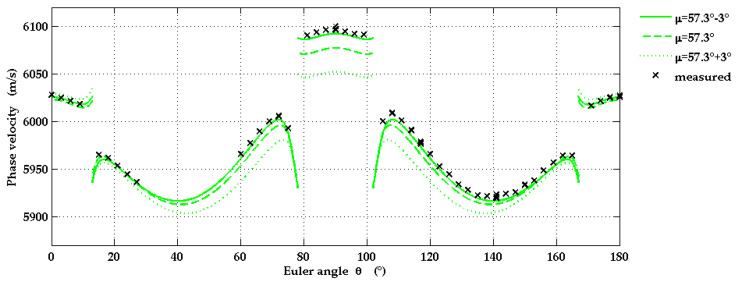
Experimental values of the SAW phase velocities for various propagation directions on r-plane sapphire (black crosses) and corresponding calculated curves, taking into account off-cut angles *χ* = ±3°. Here the elastic constants from [30] were used for calculations.

In order to find out to what accuracy the LU method is able to determine the off-cut angle *χ* of the r-plane sapphire substrate, the SAW phase velocity was calculated as a function of wavevector direction for the values *χ* = −3°, 0°, +3°. A comparison of the calculated data with the measured SAW phase velocities in Figure 7 proves that an offset angle of ±3° is clearly detectable. It also reveals in which regions of wavevector directions the phase velocity is particularly sensitive to the off-cut. The wavevector directions around *θ* = 90° are particularly suitable for the detection of off-cut angles larger or equal to 3°, whereas directions with *θ* near 0° seem to be more suitable for values of the off-cut angle smaller than −3°. However, the detected signals of the SAW pulses were comparatively weak for *θ* near 0°.

**Figure 8:**
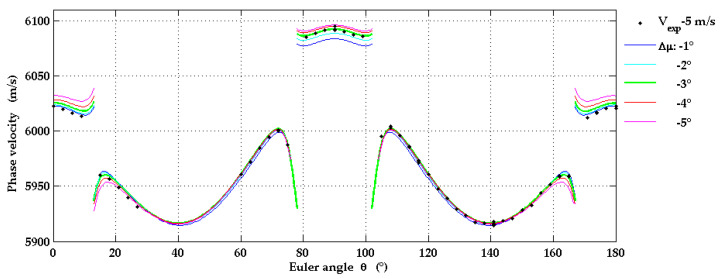
Influence of the off-cut angle *χ* on the angular dependence of the SAW phase velocity on Al_2_O_3_(1-102). Here, the experimental results are downshifted by 3 m/s.

Figure 8 shows calculated SAW phase velocities for the correct off-cut angle *χ* = −3° and four additional values of *χ*. The experimental data for the phase velocities shown in this figure are downshifted by 3 m/s as an attempt to correct a systematic error in our measurements due to a misalignment between the pump beam and the moving direction of the translation stage mentioned in Section 2.3.

The comparison of the data in Figure 8 suggests that the off-cut angle of the r-plane geometry can be determined by LU with an accuracy of almost 1° with an optimized alignment of the optical and mechanical components. Obviously the accuracy reachable in the determination of the off-cut angle by LU strongly depends on the precision to which the elastic constants and the density of sapphire are known. A 0.1 % change of the mass density translates into a phase velocity shift of approximately 3 m/s.

We note here that the SAW slowness curve on the r’-plane in Al_2_O_3_ (Euler angles (0°, 57.6°, *θ*)) differs substantially from that on the r-plane (Euler angles (60°, 57.6°, *θ*)), because the elastic constant *c*_14_ has a non-negligible magnitude in comparison to the other elastic constants (Table 3). In the case of hexagonal symmetry, the two planes would be equivalent [35]. On the r’-plane, the SAW slowness curve does not cross an intersection curve of the bulk wave slowness surface with the crystal surface. Therefore, r-plane and r’-plane samples can easily and quickly be distinguished by an LU measurement. 

The correct sign of *c*_14_ has been clarified by ab initio calculations and new experiments ([27,28] and references therein). A change of sign of c_14_ causes an interchange of the elastic properties of r-plane and r’-plane.

### 3.2. Film on Substrate: Al_0.77_Sc_0.23_N/Al_2_O_3_

In order to acquire information about the elastic constants of AlScN films on sapphire substrates, LU measurements have been carried out for the two systems Al_0.77_Sc_0.23_N(0001)/Al_2_O_3_(0001) and Al_0.77_Sc_0.23_N(11-20)/Al_2_O_3_(1-102) (in the following called “c-plane samples” and “a-plane samples” for short, respectively, Samples 1 and 3 in Table 2).

SAW dispersion curves were simulated with the semi-analytic Greens function method. Input data for the substrate material in these calculations were the set of elastic constants from [30] with the corrected sign of *c*_14_ along with the mass density of sapphire from [31] and dielectric constants from [33] (see Section 3.1.1). 

The material constants chosen for the isotropic metal layer molybdenum are given in Section 3.1.1.

**Figure 9:**
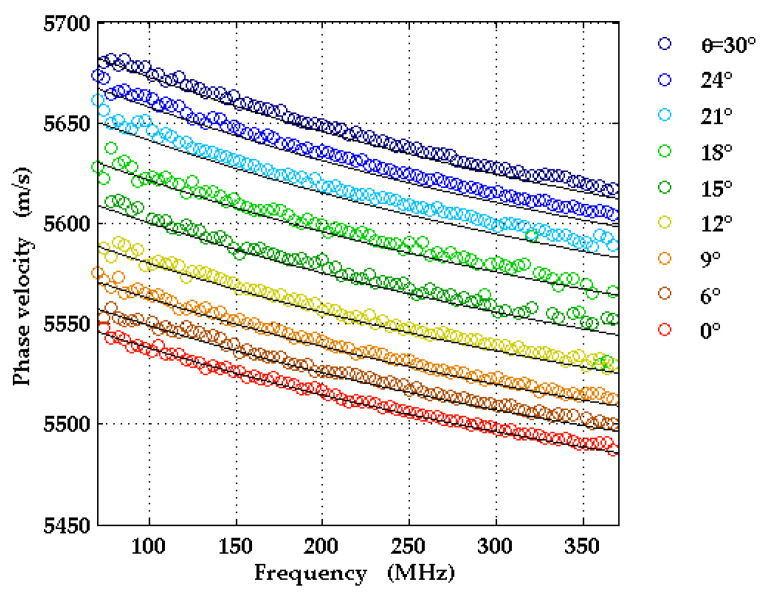
Experimental and theoretical dispersion curves for the different directions *θ* for Ti/Al_0.77_Sc_0.23_N(0001)/Al_2_O_3_(0001). In the calculations, the elastic constants from [30] for sapphire and material constants from [5] for AlScN were used.

**Figure 10:**
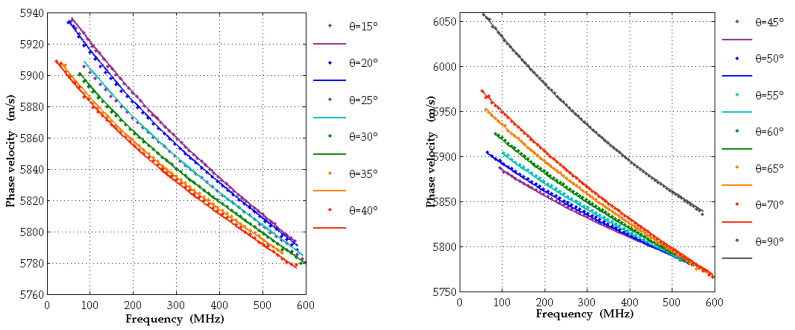
Experimental (dots) and theoretical (lines) dispersion curves for various SAW propagation directions for Mo/Al_0.77_Sc_0.23_N(11-20)/Al_2_O_3_(1-102). For the calculations the constants from [5,30] and were used.

For the elastic and piezoelectric constants of the Al_0.77_Sc_0.23_N films, we used the data obtained in ab initio calculations, based on the density functional theory, by Urban et al. [5]. In [5], an interpolation formula is given for the mass density and for each individual elastic constant *c*_μν_(x) and piezoelectric constant *e*_i__μ_(x) of Al_1-x_Sc_x_N containing terms of up to second order in the scandium concentration x. The coefficients in these formulas were obtained by a fit to the corresponding material constants from ab initio calculations for a set of scandium concentrations x. As a second variant, Urban et al. suggest a rescaling of their interpolation formulas such that in the limit x to zero, the material constants take the values calculated for pure AlN. The SAW dispersion curves simulated with these two variants of material constants for the Al_0.77_Sc_0.23_N films are indistinguishable on the scales of the graphs in Figure 6, Figure 9 and Figure 10.

For both independent dielectric constants $\varepsilon_{11}$, $\varepsilon_{33}$ the value 17.56 was used in the calculations. It follows from an interpolation formula of experimental data for $\varepsilon_{33}$, provided in [36] (see also [10]), applied to the scandium concentration x = 0.23.

#### 3.2.1. C-Plane Samples

The symmetry of the c-plane geometry implies that [0°, 30°] is an irreducible interval of angles *θ* for the SAW wavevector directions. LU measurements were taken for nine different wavevector directions corresponding to angles *θ* in this interval. For each direction, the SAW pulse shapes were recorded at 32 different distances from the source. From these data, the SAW dispersion curves were determined in a frequency range from 50 up to 400 MHz, shown in Figure 9. They exhibit a small, but non-negligible curvature. 

Simulated dispersion curves for the nine wavevector directions are also presented in Figure 9 for comparison. 

#### 3.2.2. A-Plane Samples

Because of the lower symmetry of this configuration, an irreducible interval of SAW wavevector directions on this surface is the range of angles *θ* between 0° and 90°. In Figure 10, experimental dispersion curves are shown for wavevector directions with angles *θ* from 15° to 70° in steps of 5° and, in addition, for *θ* = 90°. For angles in the vicinity of 10° or 80°, the detected signals are very weak. The reason is presumably that here the surface displacements in the direction normal to the surface are much smaller than for other wavevector directions and the same frequency, since the SAW penetrates deeply into the substrate. Here, two very weak signals emerge because of mode repulsion (see Section 3.1.1) instead of one strong signal, leading to inaccuracies in the data processing.

The dispersion curves show a modest amount of curvature which is favorable for the extraction of elastic constants of the AlScN film. The experimental dispersion curves are in good agreement with the results of calculations, carried out with input data from [5]. 

### 3.3. Sensitivity Analysis

**Figure 11:**
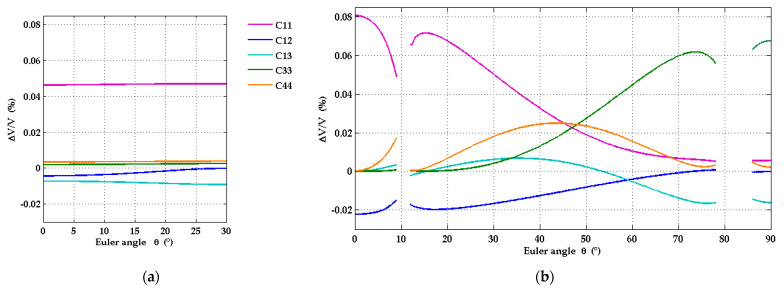
Calculated relative change of phase velocity with 1% increase of elastic constants for Mo/Al_0.77_Sc_0.23_/Al_2_O_3_ structures with 1 µm thick Al_0.77_Sc_0.23_N film and additional Mo coating with 50 nm thickness, as a function of SAW propagation direction on the surface. (**a**) case of AlScN(0001)/Al_2_O_3_(0001) and (**b**) AlScN(11-20)/Al_2_O_3_(1-102).

In order to assess to what extent the agreement between the dispersion curves measured with the LU method and those simulated with the calculated material constants of [5] can be taken as a confirmation of the latter, the sensitivity of the dispersion curves with respect to changes of each single elastic constant of the AlScN film has been analyzed (Figure 11). With the same material data used for the simulation of the dispersion curves in Figure 9 and Figure 10, we calculated the relative change Δ*v/v* of phase velocity *v* with a 1% increase for each of the five independent elastic constants *c*_μν_ (see Table 3), while leaving the remaining material constants unchanged. Figure 11a,b show results of this calculation for the c-plane geometry and the a-plane geometry, respectively. Δ*v/v* is plotted as a function of wavevector direction at the fixed frequency 400 MHz. This frequency value has been chosen since it corresponds to the upper edge of the frequency interval of the measured dispersion curves for the c-plane samples and is located in the upper third of the frequency band for the a-plane geometry. With increasing frequency, the fraction of the SAW displacement field localized in the AlScN film is expected to rise as well. When comparing the relative velocity changes in Figure 11a,b, one has to account for the slightly different thicknesses of AlScN layer in the two types of samples (1 µm for the c-plane, 860 nm for a-plane samples). 

In the case of the c-plane samples, the sensitivities of the SAW phase velocity with respect to changes in the elastic constants of the AlScN film are comparatively small, except for the constant *c*_11_. The isotropy of the hexagonal film in the surface plane and the moderate deviations of the SAW slowness curve from a circle on c-plane sapphire (Figure 3e) are reflected in a very weak dependence of the sensitivities with respect to the wavevector direction (Figure 11a), with the exception of the sensitivity to changes of *c*_12_, which vanishes at the wavevector direction with Euler angle *θ* = 30°.

In the case of the a-plane geometry, the sensitivities exhibit a remarkable dependence on the wavevector direction. This is due to the strong anisotropy of the film in the surface plane, and it is also associated with the strong variation of the SAW mode pattern in the neighborhood of the Euler angles *θ*, where the SAW slowness curve of r-plane sapphire crosses the intersection curve of the slowness surface of acoustic bulk waves. Moreover, the sensitivities are on average clearly larger than those on the c-plane samples. 

**Figure 12:**
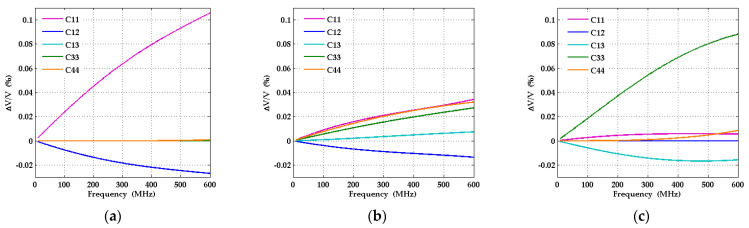
Relative change of phase velocity with 1% increase of elastic constants in the structure AlScN(11-20)/Al_2_O_3_(1-102) with 1000 nm thick Al_0.77_Sc_0.23_N film and 50 nm molybdenum film as function of frequency for the cases (**a**) *θ* = 0°; (**b**) *θ* = 45°; (**c**) *θ* = 90°.

A feature of particular interest is the relative size of the sensitivities for the directions with Euler angles *θ* in the vicinity of 0° on the one hand and in the neighborhood of 90° on the other. In the first range of wavevector directions, the sensitivities with respect to *c*_11_ and *c*_12_ dominate, while the sensitivities with respect to the other elastic moduli are largely negligible. In the second range, the dispersion curve is mainly sensitive to *c*_33_ and *c*_13_ and the other elastic constants play a largely negligible role. Knowledge of this behavior should be very helpful for fitting strategies to extract the elastic constants from measured dispersion curves. At wavevector directions with Euler angles *θ* around 45°, the relative velocity variations with relative changes of *c*_11_, *c*_33_, and *c*_44_ are of comparable size. In general, one may notice that for each elastic constant there are ranges of wavevector directions where this constant has a non-negligible influence on the SAW dispersion curves. Figure 12 shows how the sensitivities vary as functions of frequency for the fixed wavevector directions with Euler angles *θ* = 0°, 45° and 90°. The sensitivities with respect to almost all elastic constants increase at higher frequencies because of the increasing localization of the SAW displacement field in the AlScN film. In the case of *θ* = 0°, the sensitivities with respect to *c*_11_ and *c*_12_ dominate over the whole frequency range from zero to 600 MHz, and likewise the sensitivities with respect to *c*_33_ and *c*_13_ in the case of *θ* = 90°.

The sensitivities of the dispersion curves for SAW in AlScN films on sapphire, discussed above, may be compared with those for a c-plane Al_0.68_Sc_0.32_N film on a Si(001) substrate, presented in [17] for two different wavevector directions. (Note that the data in Figure 3 of [17] refer to a relative change of 10% of the elastic and piezoelectric constants. The thickness of the AlScN(0001)/Si(001) was ~1 µm.) Remarkably, the sensitivities for the c-plane film on silicon are of comparable size to those of the a-plane sample. However, in both wavevector directions in the c-plane film on the silicon substrate, the influence of the elastic constant *c*_11_ of AlScN dominates. 

Figure 3b in [17] clearly shows that the sensitivities of the SAW phase velocities with respect to the piezoelectric constants (from D. Urban et al. in Reference [5]) are by more than one order of magnitude smaller than the ones with respect to the elastic constants.

## 4. Discussion and Conclusions

The main results of the investigations reported in this contribution may be summarized as follows:

- SAW dispersion curves were measured by laser ultrasound for various wavevector directions in c-plane and a-plane Al_0.77_Sc_0.23_N films on sapphire substrates. They are in very good agreement with the corresponding theoretical dispersion curves computed with the elastic moduli and piezo-electric constants obtained in ab initio calculations by Urban et al. [5]. The theoretical elastic constants for Al_1-x_Sc_x_N in [5] are given in the form of interpolation formulas quadratic in the parameter x. For the Sc concentrations, x = 0.14 and x = 0.32, the authors of [5] compare their calculated elastic constants with corresponding data determined by Kurz et al. [10] with the help of SAW resonators. For both concentrations, the agreement is very good (2% deviation on average, less than 5% in the worst case). This confirms the high-quality of the theoretical data in [5] and may also be regarded as an additional, indirect confirmation of the measured SAW dispersion curves presented here.
- The off-cut angle of 3° in the r-plane sapphire substrate, needed to provide optimal growth conditions for a-plane AlScN, can easily be detected with the laser ultrasound setup described above.
- Sensitivities of the SAW dispersion curves with respect to variations of each of the independent elastic constants of the AlScN film have been calculated as functions of the SAW wavevector direction and frequency. Due to the strong anisotropy of the a-plane geometry in the surface plane, the sensitivities show strong variations with wavevector direction. In certain intervals of wavevector directions, only a few elastic constants predominantly influence the dispersion curve, while the sensitivity with respect to the others is much smaller. In other wavevector directions, certain elastic constants change their role with respect to their influence on the SAW dispersion curves. This finding can be made use of in the development of fit strategies for an accurate determination of all elastic constants of AlScN films.

The size of the sensitivities at around 400 MHz leads us to the following conclusion. Assuming that a relative change of velocity by 6 m/s is resolvable in the LU experiment, a change of 2% in the elastic constants *c*_11_ of *c*_33_ of the AlScN should be detectable. With an efficient use of the anisotropy of both the substrate and the film and with sufficiently small attainable SAW wavelengths on the scale of the film thickness (i.e., a sufficiently large frequency range), a determination of the elastic constants of AlScN films should be achievable. Even in the long-wavelength limit, when the dispersion curves are essentially straight lines, certain combinations of material constants can be extracted.

If the minimal achievable SAW wavelength is larger or of the order of the film thickness, such that the SAW displacement field penetrates deeply into the substrate, the elastic properties of the substrate as well as its orientation (e.g., off-cut angles) have to be known to a high precision. On the other hand, if the frequency range can be considerably extended to higher values, higher-order guided acoustic modes can be used in addition to the lowest SAW to gain information about the elastic moduli of the film [11]. 

The sensitivity analysis in [17] confirms that the piezoelectric constants have a very small influence on the SAW dispersion curves if compared to the elastic constants. However, depending on the required accuracy in the determination of the elastic moduli, auxiliary measurements with an alternative technique, such as the resonator method [10,11] will be needed. 

The measured SAW dispersion curves for c-plane and a-plane Al_0.77_Sc_0.23_N compare very favorably with dispersion curves calculated with the data for the elastic and piezoelectric constants in [5] for all SAW wavevector directions. These data were obtained in ab initio calculations treating the atomic positions as static, which means that phononic thermal contributions are disregarded. Very rough estimates on the basis of data for the temperature dependence of Young’s modulus of AlN ceramics [37] and predictions of the temperature dependence of the elastic moduli of AlN with highly simplifying assumptions [38] suggest that the thermal contributions at room temperature to the elastic moduli are smaller than 1% of their total value. The sensitivities of the SAW dispersion curves, discussed above, imply that such small variations cannot be resolved with our current laser ultrasound setup. 

In conclusion, the results for AlScN films on sapphire substrates, presented in this work, confirm that laser ultrasound can be applied as a viable tool for the determination of elastic properties of anisotropic, including piezoelectric films on anisotropic substrates. This requires measurements of SAW dispersion curves for various propagation directions, which can be pre-selected by simulations and a detailed sensitivity analysis. In comparison to isotropic films, anisotropy poses an additional challenge because of an increased number of independent elastic constants. At the same time, the anisotropy of the film and of the substrate offers the possibility of gaining additional information on different elastic constants from certain different SAW propagation directions.

## Figures and Tables

**Table 1:** Growth parameters of Al_0.77_Sc_0.23_N.

Parameter	Value
N2 gas flow (sccm)	20 ^1^, 30 ^2^
Target-to-substrate distance (mm)	65
Chuck temperature (°C)	450
Base pressure (Pa)	<9 × 10^−6^
P_Al_ + P_Sc_ (W)	1000

^1^ for AlScN(0001)/Al_2_O_3_(0001). ^2^ for AlScN(11-20)/Al_2_O_3_(1-102)

**Table 2:** Samples investigated in this work.

Material	Sample 1	Sample 2	Sample 3
Substrate	Al_2_O_3_(0001)	Al_2_O_3_(1-102)	Al_2_O_3_(1-102)
Piezoelectric film, thickness (nm)	Al_0.77_Sc_0.23_N(0001)	-	Al_0.77_Sc_0.23_N(11-20)
	1035	-	860
Metal film, thickness (nm)	Mo	Mo	Mo
	50	50	50

**Table 3:** Elastic constants *c*_μν_ for sapphire, GPa.

	**Tefft et al.** **1966** [29] *	**Gieske et al.** **1968** [30]	**Goto et al.** **1989** [31]	**Landolt-** **Börnstein** [32]	**Auld** **1990** [33]	**Gladden** **2004** [27]
*c*_11_	497.4	497.6	497.3	496	494	497.5
*c*_12_	164.0	162.6	162.8	159	158	162.7
*c*_13_	112.3	117.2	116	114	114	115.5
*c*_14_	−23.6	−22.9	−21.9	−23	−23	22.5
*c*_33_	499.4	501.9	500.9	499	496	503.3
*c*_44_	147.4	147.2	146.8	146	145	147.4

* Calculated from compliances. In all calculations we took the same density *ρ* = 3982 kg/m^3^ and dielectric permittivities $\varepsilon_{11}$ = 9.34 and $\varepsilon_{33}$ = 11.54 from [33].

## Data Availability

Data are available from the corresponding authors upon reasonable request.
